# Supplementary material for: Molecularly Targeted Photothermal Ablation of Epidermal Growth Factor Receptor-Expressing Cancer Cells with a Polypyrrole–Iron Oxide–Afatinib Nanocomposite
Source: Cancers (Basel). 2022 Oct 14;14(20):5043. doi: 10.3390/cancers14205043 (PMC9599920; doi:10.3390/cancers14205043)
Supplement: Supplementary file 1 [file cancers-14-05043-s001.zip › cancers-1952872-supplementary.pdf]

SUPPLEMENTARY DATA

Materials and methods

Synthesis of PPy NPs: Ppy Nps was synthesized using a modified protocol of a previously established procedure.[10] 0.05 M pyrrole was dissolved in 80 mL water, then heated to 55°C for 15 min after adding 16 mL of 0.09 M Ferric Chloride, and 2400 μL of 0.4 M polyethyleneimine was added to the solution drop by drop. The solution was retained at room temperature (RT) overnight to ensure enough stirring. The solution was dialyzed for 24 h in the 3500 MWCO dialysis bag.

Synthesis of IO NPs*:* A chemical precipitation approach was used to make IO NPs from Ferrous Chloride and Ferric Chloride. In a 1:2 molar ratio, Ferrous Chloride and Ferric Chloride were added. The water was preheated to 60°C and under vigorous stirring, $FeCl_3$ (5 mmols) and $FeCl_2$ (2.5 mmols) were combined.3M NaOH was added drop by drop, to the aqueous solution until it turned black at RT. The generation of IO (Fe_3O_4) NPs was indicated by the color-changing to black. After the formation of the IO NPs, stirring was continued at RT for another 30 min. Magnetic separation was used to recover the NPs, and deionized water was used to rinse away the pollutants.

Synthesis of PI NPs: The PI NPs were synthesized using a chemical precipitation method. There is an electrostatic interaction between the positively charged PEI- PPy and the negatively charged IO. 0.05 M pyrrole was dissolved in 80mL water. After adding 16mL of 0.09M Ferric Chloride that had been prepared ahead of time, the solution was heated to 55°C for 15 minutes. 2400μl of 0.4 M polyethyleneimine was added to the solution drop by drop. 10 mg of an aqueous solution of IO is added after the PEI has been added. After being agitated overnight, the solution was dialyzed for 1 day using the 3500MWCO membrane.

Invitro studies

ROS generation by PIA NC

The A549 cells were cultured at a cell density of 1×10^5 in the dish and incubated for 24 h. The cells were washed using PBS before being exposed to IO NPs in DMEM. Cells were exposed to NIR irradiation (2.45 W/cm2 at 808 nm) for 1 minute and without NIR irradiation after the NC treatments. Followed by PBS washing the DCFH-DA is used to stain (ROS staining) and incubated for 30min. The cells were washed twice with PBS solution. After lysating the cells were centrifuged at 2300g for 10min. Then 250μl supernatant was transferred to 96 well plate and fluorescence was measured using microplate reader. (485 nm excitation and 520 nm emission)

Results

Characterization of NC’s

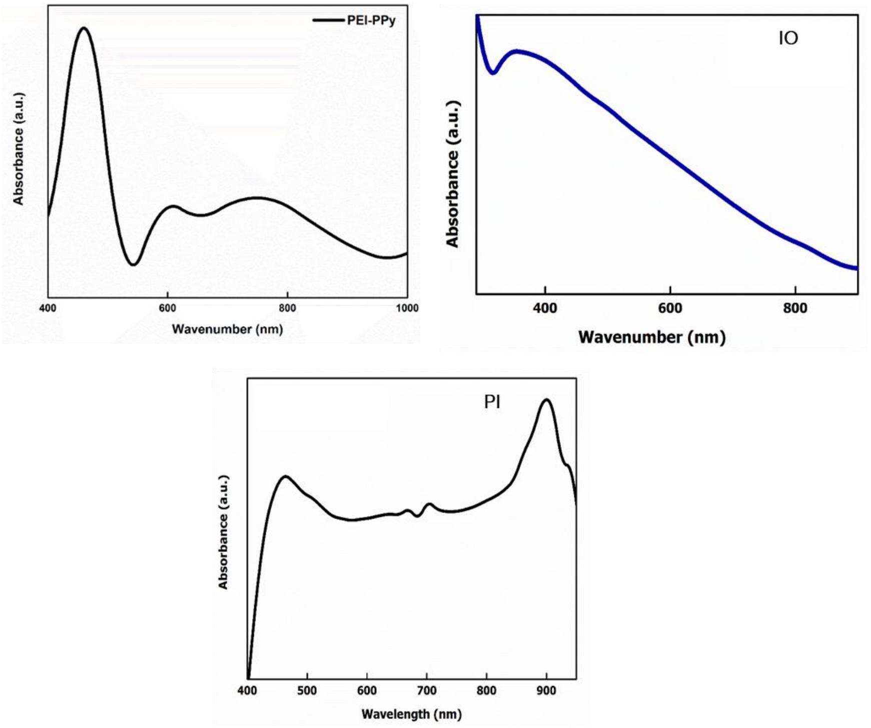

Figure S1: UV data of (A) Ppy, (B) IO, (C) PI respectively.

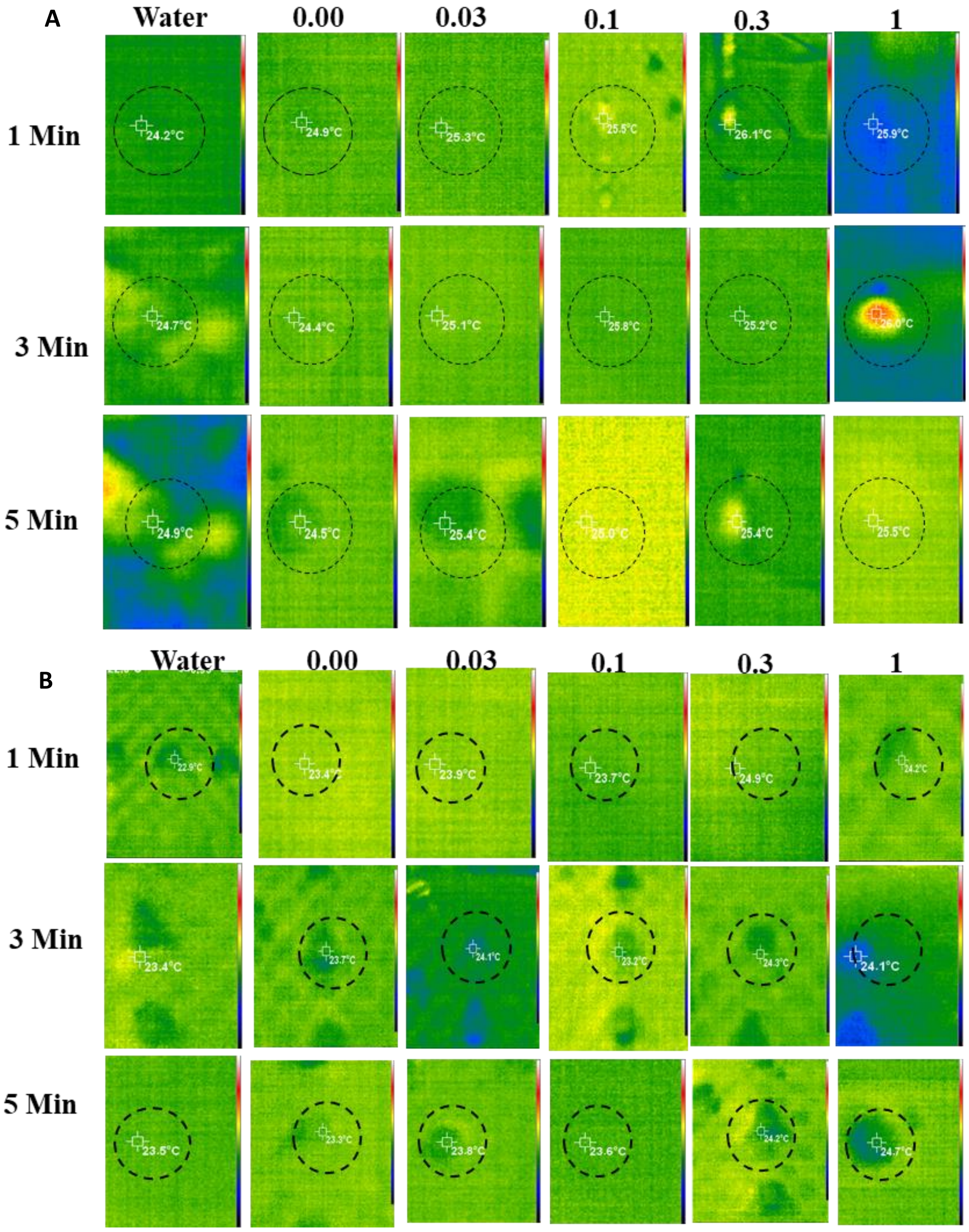

Figure S2: Thermal camera images of PIA irradiated with (A) UV and (B) Visible light at different concentration and time.

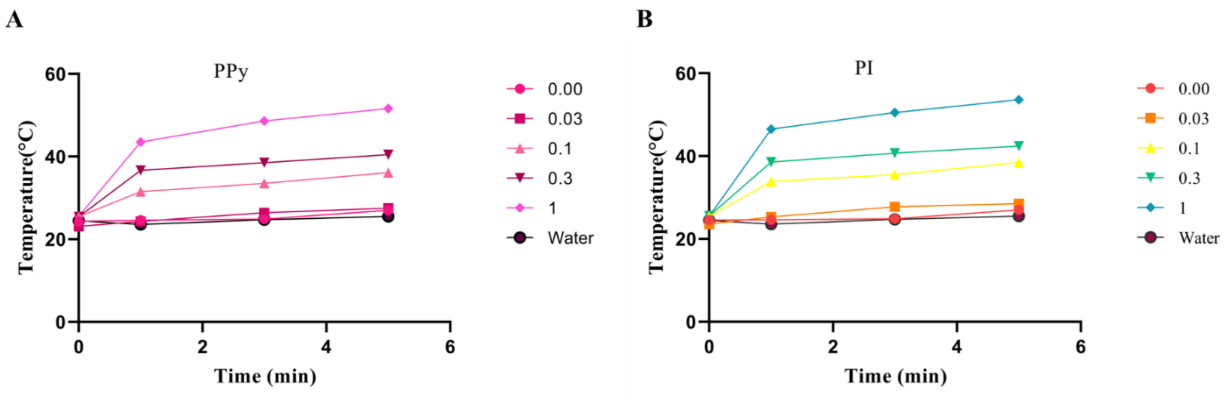

Figure S3: PTT graph (A) PPy and (B) PI.

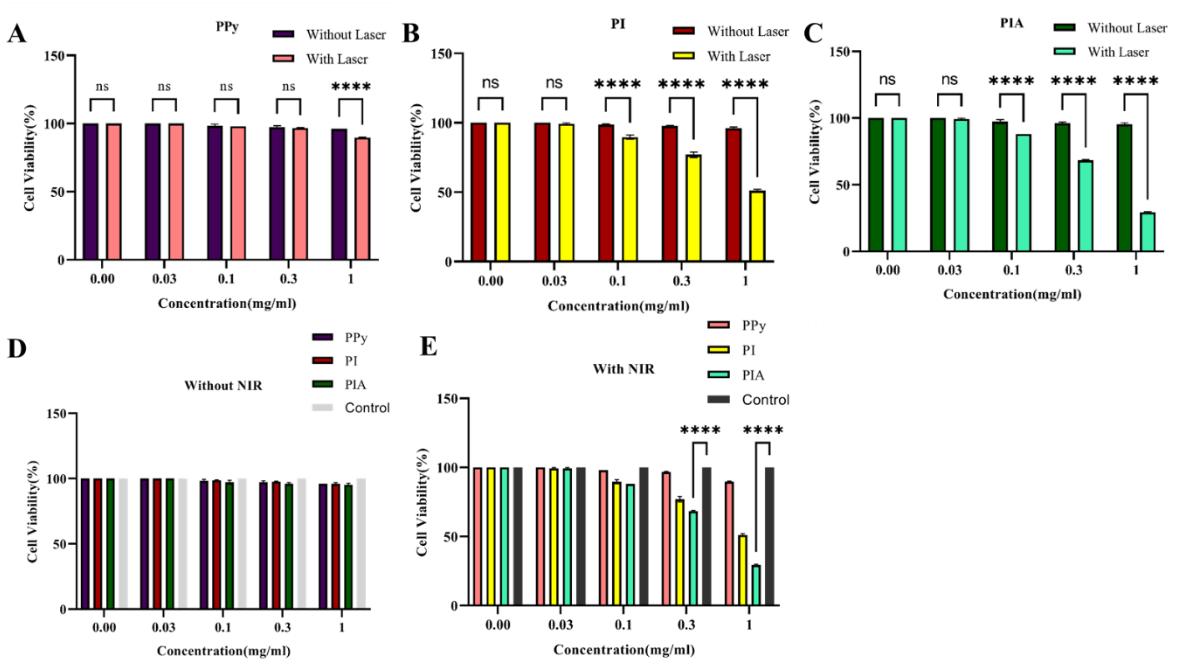

Figure S4: Cytotoxicity data of (A) Ppy, (B) PI and (C) PIA with and without NIR irradiation. Comparison of the Ppy, PI and PIA (D) without and (E) with NIR irradiation. **** p value < 0.0001.

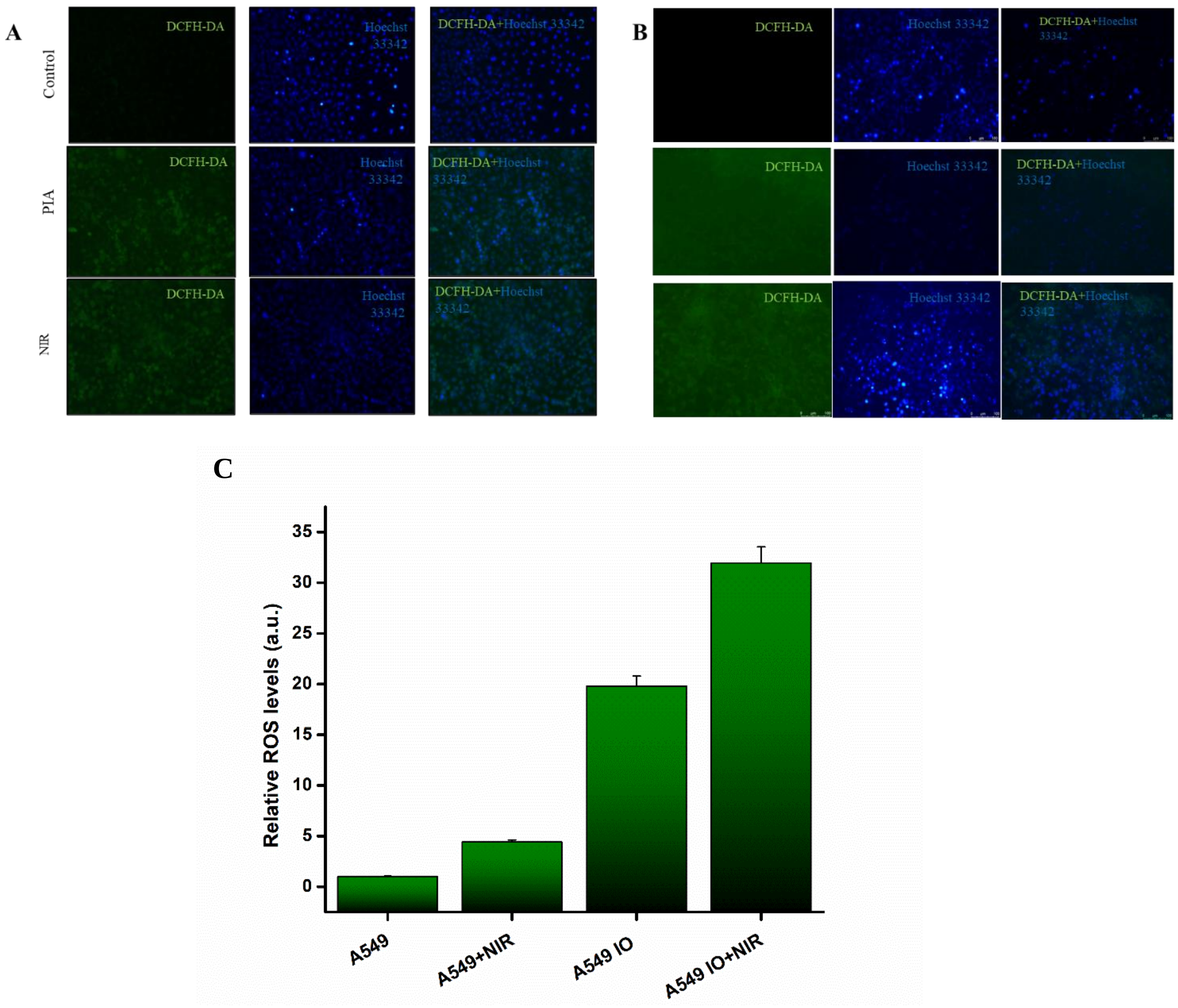

Figure S5: The ROS production by (A) A549 cells and (B) NIH3T3 cells after PIA and NIR treatment. (C) ROS generation by IO in A549 with and without NIR.

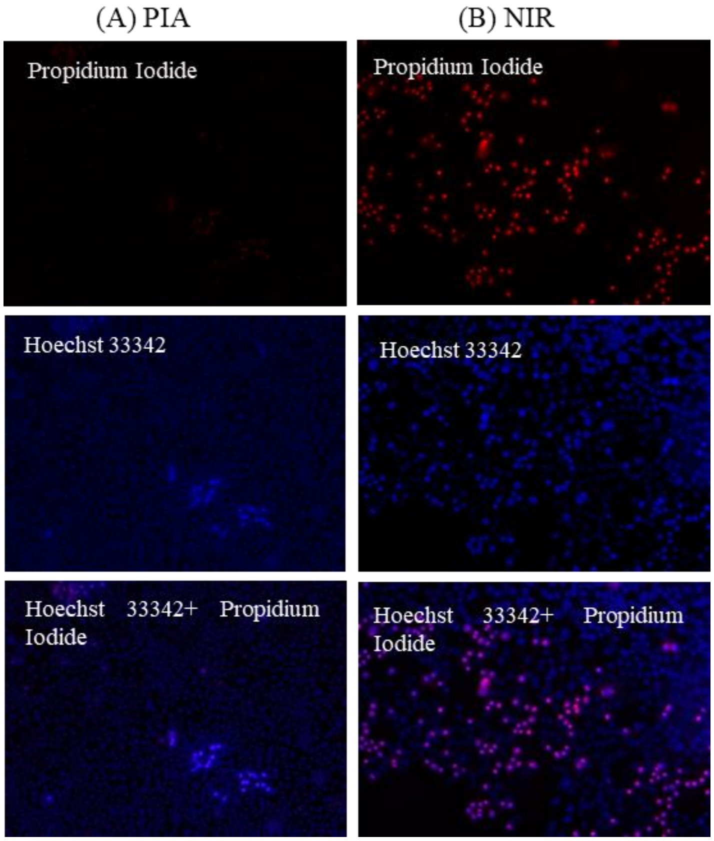

Figure S6: The fluorescent microscopy analysis of the apoptosis in A549 cells under (A) PIA and (B) NIR treatment alone.
